# Live Discharge From Hospice: Experiences of Patients, Families, and Staff

**DOI:** 10.1093/geroni/igaa057.2231

**Published:** 2020-12-16

**Authors:** Rebecca Utz, Margaret Clayton, Katherine Supiano

**Affiliations:** 1 University of Utah, Salt Lake City, Utah, United States; 2 University of utah, Salt Lake City, Utah, United States

## Abstract

Hospice services can provide patient stabilization and improved quality of life, making the patient ineligible for continued hospice. Using focus groups and in-depth interviews, we explored how patients, families, and hospice staff experience, anticipate, and cope with “live discharge” from hospice. Nurses and administrators tried to prepare families for the possibility and unsettling reality of live discharge, yet the aides who worked most closely with families remained unaware of regulations dictating an impending live discharge. Reactions of families ranged from excitement, when patient was selected for clinical trial, to more common expressions of frustration, as they lost access to the staff, coordinated services, and medications only covered under the Medicare hospice benefit. Families also reported relationship stressors and decisional uncertainty associated with longer than expected patient life. Families still needed and wanted hospice services, with most agreeing they would return to hospice after the patient declined and became eligible again.

